# Advancements in research on the cardiovascular toxicity caused by TEC family kinases inhibitors

**DOI:** 10.3389/fphar.2025.1726458

**Published:** 2026-01-06

**Authors:** Yi Zhang, Huangxi Fu, Xingchen Kang, Zixuan Qiu, Hao Yan, Qiaojun He, Bo Yang, Zhifei Xu, Peihua Luo

**Affiliations:** 1 Center for Drug Safety Evaluation and Research of Zhejiang University, College of Pharmaceutical Sciences, Zhejiang University, Hangzhou, Zhejiang, China; 2 Innovation Institute for Artificial Intelligence in Medicine of Zhejiang University, Hangzhou, Zhejiang, China; 3 School of Medicine, Hangzhou City University, Hangzhou, China; 4 Institute of Pharmacology and Toxicology, Zhejiang Key Laboratory of Anti-Cancer Drug Research, College of Pharmaceutical Sciences, Zhejiang University, Hangzhou, China

**Keywords:** bruton’s tyrosine kinase, cardiovascular toxicity, clinical safety, inhibitors, TEC family kinases

## Abstract

The tyrosine kinase expressed in hepatocellular carcinoma (TEC) family kinases (TFKs) are a subfamily of non-receptor protein tyrosine kinases (PTKs) that include five members: TEC, bruton’s tyrosine kinase (BTK), interleukin 2-inducible T-cell kinase (ITK/EMT/TSK), bone marrow tyrosine kinase on chromosome X (BMX/ETK), and tyrosine-protein kinase (TXK/RLK). They play key roles in cell signaling and immune regulation. Emerging evidence highlights their involvement in cardiovascular diseases (CVDs) such as ischemic heart disease (IHD), atherosclerosis (AS), sepsis-related dysfunction, atrial fibrillation (AF), myocardial hypertrophy, coronary atherosclerotic heart disease, myocardial infarction (MI), and post-myocardial infarction complications. However, no review has comprehensively addressed the cardiovascular toxicity of TFKs inhibitors. This review provides a comprehensive and systematic analysis of the cardiovascular toxicity profiles of TFK inhibitors (TFKis), focusing on underlying molecular mechanisms, comparing toxicity across different agents and generations, and discussing clinical implications.

## 1 Introduction

Protein kinases catalyze the phosphorylation of proteins, altering their activity or their ability to interact with other molecules, thereby affecting cellular growth, differentiation, survival, and proliferation. Additionally, kinases play a role in numerous signal transduction cascades. Thus, the dysregulation of protein kinase activity is pivotal in the pathogenesis of numerous diseases, encompassing autoimmune, cardiovascular, neurological, and inflammatory disorders, as well as a spectrum of cancers (Das and Hong, 2020). TEC family kinases (TFKs), a subgroup of the non-receptor tyrosine kinases (NRTKs), constitute the second-largest family of cytoplasmic tyrosine kinases in humans. Receptor-mediated signaling is known to be highly complex, guiding many parallel processes such as proliferation, programmed cell death, differentiation, migration, and secretion. Together, these processes underpin complex and essential behaviors of the organism (Yu and Smith, 2011). Therefore, TFKs play significant roles in various cellular processes, including immune responses, cell survival, and signaling pathways involved in cancer, inflammation, and CVDs (Yin et al., 2022). TFKs, such as TEC, BTK, and ITK, have been identified as therapeutic targets of autoimmune disorders and cancers (Yin et al., 2022).

However, the clinical use of TFK inhibitors (TFKis), particularly Bruton’s tyrosine kinase inhibitors (BTKis), has revealed significant cardiovascular adverse effects, such as AF, hypertension, and heart failure (HF). Although several reviews have focused on BTKi cardiotoxicity, a comprehensive synthesis encompassing all TFK members (BTK, TEC, ITK, BMX, TXK) is lacking. This review aims to fill this gap by systematically examining the cardiovascular toxicity profiles, mechanistic insights, and clinical management strategies associated with TFKis, thereby providing a holistic perspective to inform clinical practice and future drug development.

## 2 Overview of TEC family kinases and their inhibitors

### 2.1 TEC family kinases members

TEC family kinases (TFKs), the second largest subfamily of the NRTKs, consist of five members, including TEC, BTK, ITK/EMT/TSK, RLK/TXK and BMX/ETK (Siveen et al., 2018). TEC was initially identified in liver cancer cells and is widely expressed in hematopoietic cells, playing a crucial role in immune cell signaling. TEC is involved in regulating the activation of various immune cells, including T cells and B cells (Lucas et al., 2003; Yin et al., 2022). BTK is a well-known TFK critical for B cell development and signaling. Mutations in BTK result in X-linked agammaglobulinemia (XLA), a condition characterized by a severe deficiency in B cell function and immunodeficiency (Marron et al., 2012). ITK is primarily expressed in T cells and plays a vital role in T cell receptor signaling, affecting T cell activation, differentiation, and immune response (Lechner et al., 2020). Like other TFK, TXK is involved in immune cell signaling, specifically within the T cell lineage. BMX is a TFK implicated in hematopoiesis and immune responses, especially within T cells and NK cells (Yin et al., 2022).

### 2.2 Structure and function of TEC family kinases

**FIGURE 1:**
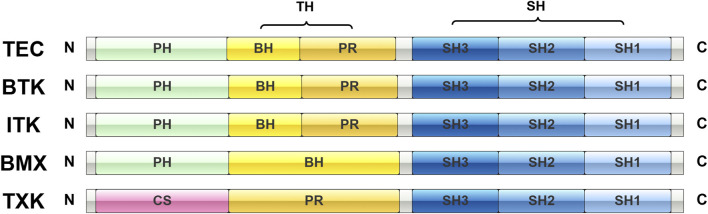
Schematic representation of TEC family kinases and their domains. PH, pleckstrin homology; CS, cysteine; BH, Btk homology; PR, proline-rich; TH, Tec homology; SH, Src homology.

TFKs share a conserved C-terminal kinase domain and an N-terminal region lacking a transmembrane helix (Figure 1) (An and Zhang, 2024; Yang et al., 2001). At their amino terminus, all members of this family except TXK contain a pleckstrin homology (PH) domain. As PH domain can bind phosphoinositides, TFKs are assumed to act as the connection between phosphotyrosine-mediated and phospholipid-mediated signaling pathways; for BTK, this domain has been shown to bind inositol phosphates *in vitro* and to be responsible for membrane localization *in vivo* (An and Zhang, 2024). In TXK, the PH structural domain is replaced by a cysteine (Cys) module that facilitates membrane association through palmitoylation (An and Zhang, 2024). TFKs also share a TEC homology (TH) domain (An and Zhang, 2024; Yang et al., 2001) containing a series of zinc-binding BTK homology (BH) modules and proline-rich (PR) modules (An and Zhang, 2024). However, the TH domain of TXK lacks the BH module, while the TH domain of BMX lacks the PR module (An and Zhang, 2024). The TH domain is sequentially followed by the Src Homology 3 (SH3), Src Homology 2 (SH2), and Src Homology 1 (SH1) domains (Andersen et al., 2019; Yang et al., 2001).

The SH3 domain primarily facilitates protein-protein interactions and has been found to potentially play a role in the negative regulation of protein tyrosine kinase activity (Andersen et al., 2019). Studies on BTK, ITK, and TXK have shown that their autophosphorylation sites are located in the SH3 structural domain (An and Zhang, 2024). The SH2 domain is responsible for mediating interactions with phosphorylated tyrosine residues on other proteins, essential for signal transduction (An and Zhang, 2024). Kinase domain that mediates phosphorylation of downstream targets, typically involved in cellular signaling pathways. PH domain interacts with phosphoinositides and helps regulate the localization of the kinase within the cell (Yin et al., 2022). These domains allow TFKs to transduce signals in response to various stimuli, including antigen receptor engagement and cytokine signaling.

## 3 Mechanism of cardiovascular toxicity induced by TEC family inhibitors

### 3.1 BTK inhibitors (BTKis)

#### 3.1.1 Ibrutinib (Imbruvica®)

Ibrutinib was the first BTKi approved by the FDA in 2013 (Table 1). It is an orally administered, effective, irreversible first-generation BTK inhibitor that covalently binds to a cysteine residue (Cys-481) near the ATP-binding pocket of BTK (Honigberg et al., 2010). As a result, it inhibits B cell receptor (BCR) signaling and downregulates nuclear factor kappa-B (NF-κB) signaling, drastically decreasing tumor growth and boosting apoptosis in the process. Ibrutinib is used to treat chronic lymphocytic leukemia (CLL), small lymphocytic lymphoma (SLL), Waldenström’s macroglobulinemia (WM), marginal zone lymphoma (MZL), and graft-versus-host disease (GVHD) (Christensen et al., 2022; Honigberg et al., 2010).

**TABLE 1:** Research status of BTK inhibitors launched in the market.

Drug	Characteristic(s)	Disease(s)	R&D progress and R&D company	IC50 (nM)					References(s)
				BTK	ITK	TEC	BMX	TXK	
Ibrutinib/Imbruvica (PCI-32765)	First-in-class BTKi	Treated or untreated CLL/SLL (2014), WM (2015), cGVHD (2017)	Approved by FDA and EMA in 2013 Pharmacyclics/Abbvie/Johnson and Johnson	0.5	10.7	78	0.8	2	Honigberg et al. (2010), Cameron and Sanford (2014), Schwarzbich and Witzens-Harig (2014)
Acalabrutinib/Calquence (ACP-196)	Decreased off-target bindings Increased selectivity Improved safety profiles	Untreated or refractory CLL/SLL, pretreated MCL	Approved by FDA and EMA in 2017 AstraZeneca/Acerta Pharma BV	5.1	>1,000	93	46	368	Byrd et al. (2016), Acalabrutinib (2012), Markham and Dhillon (2018)
Zanubrutinib/Brukinsa (BGB-3111)	Decreased off-targets with improved safety profiles Higher selectivity	CLL/SLL, untreated or pretreated MCL, previously treated or untreated WM	Approved by FDA, EMA and NMPA in 2019 BeiGene	0.22	30	1.9	36	170	Zanubrutinib (2012), Syed (2020)
Tirabrutinib/Velexbru® (GS/ONO-4059)	Decreased off-targets with increased selectivity	R/R PCNSL, treated or untreated WM, LPL	Approved by PMDA in 2020 (for use in Japan only) Ono Pharmaceutical	6.8	>20,000	48	6	92	Dhillon (2020)
Orelabrutinib (ICP-022)	Reduces off-target effects with similar selectivity to Zanubrutinib Higher synergy with R-CHOP regimen than ibrutinib	Previously treated R/R MCL, CLL/SLL, R/R MZL, MS	Approved by NMPA in 2020 (for use in China only) Approved by FDA (BTD) in 2021 Innocare Pharma	1.6	NA	NA	NA	NA	Dhillon (2021), Zhang et al. (2020)
Pirtobrutinib (LOXO-305)	First-in-class noncovalent FDA-approved BTKi	Previously treated CLL/SLL, Previously treated R/R MCL	Approved by FDA in 2023 Eli Lilly and Company	3.15	>5,000	1,234	1,155	209	Keam (2023)

CLL, chronic lymphocytic leukemia; SLL, small lymphocytic lymphoma; WM, Waldenström’s macroglobulinemia; cGVHD, chronic; MCL, mantle cell lymphoma; R/R PCNSL,relapsed/refractory primary central nervous system lymphoma; LPL, lymphoplasmacytic lymphoma; R/R MZL, relapsed/refractory marginal zone lymphoma; MS, multiple sclerosis; NA, no data.

In addition to BTK, ibrutinib inhibits other intracellular kinases, including B lymphoid tyrosine kinase (BLK), BMX, TEC, ITK, and Janus kinase 3 (JAK3) (Rozkiewicz et al., 2023). The advent of BTKi has generated a unique toxicity profile, driven by off-target inhibition of other kinases (Lipsky and Lamanna, 2020). With 11 years of data since the initial approval of ibrutinib, the toxicity profile of BTKi as a class is well established, including cardiac arrhythmias (CA), hypertension, bleeding, infections, diarrhea, and arthralgias (Dickerson et al., 2019; Guha et al., 2018; Khountham et al., 2021; Mato et al., 2018; Wiczer et al., 2017). Adverse effects, rather than disease progression, are the most common reason for discontinuing ibrutinib, with cardiac side effects causing more than 10% of patients to discontinue treatment. Atrial fibrillation (AF) is the most common cause of drug discontinuation due to toxicity in patients taking ibrutinib, leading to cessation in 20%–60% of cases where it occurs (Maddocks et al., 2015; Wiczer et al., 2017). It occurs in 5%–16% of patients, most frequently in those older than 65 years of age and/or with cardiovascular risk factors (Pineda-Gayoso et al., 2020; Tam et al., 2020; Wiczer et al., 2017). Hypertension is the most common cardiac adverse event associated with ibrutinib, reported in up to 30% of patients in clinical trials and up to 80% in real-world studies (Dickerson et al., 2019). The summary of long-term follow-up data from several late-stage irutinib trials indicates that there may be an increased risk of heart failure. In these analyses, it was observed that up to 5% of patients developed heart failure, typically occurring several years after the initiation of treatment (Barr et al., 2022; Munir et al., 2019). A large retrospective analysis of 860 patients with CLL treated with ibrutinib revealed that, compared with chemotherapy, the risk of heart failure in patients treated with ibrutinib was 7.7% over 3 years (Table 2) (Abdel-Qadir et al., 2021).

**TABLE 2:** Cardiovascular toxicities associated with BTK inhibitors.

Drug	Atrial Fibrillation	Ventricular Arrhythmias	Hypertension	Heart Failure
Ibrutinib	+++	++	+++	+
Acalabrutinib	++	++	++	?
Zanubrutinib	++	?	++	?
Pirtobrutinib	+	?	?	?

? Indicates areas where systematic cardiac data are not widely available.

**FIGURE 2:**
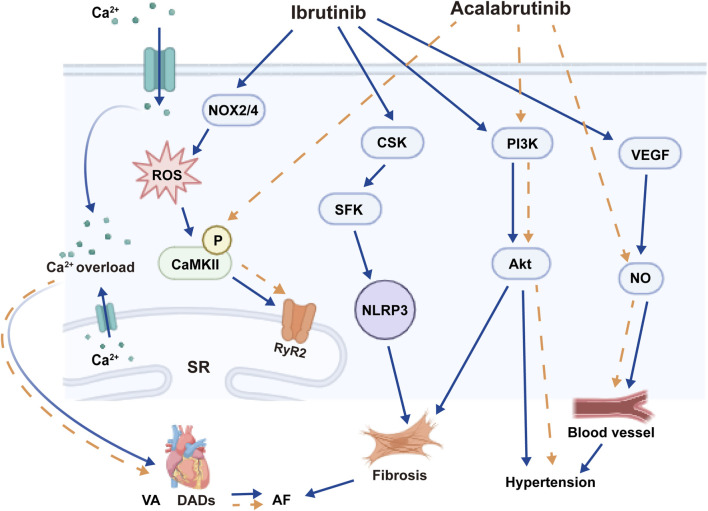
Mechanisms of Ibrutinib and Acalabrutinib-Induced Cardiotoxicity. NOX2/4,NADPH oxidase; ROS, reactive oxygen species; CaMK Ⅱ, calcium/calmodulin dependent protein kinase Ⅱ; SR, sarcoplasmic reticulum; RyR2, ryanodine receptor 2; VA, ventricular arrhythmias; DADs, delayed after depolarization; AF, atrial fibrillation; CSK, C-terminal Src kinase; SFK, Src family kinase; NLRP3, NOD-like receptor thermal protein domain associated protein 3; PI3K, Phosphatidylinositol 3-kinase; Akt, protein kinase B; VEGF, Vascular endothelial growth factor; NO, Nitrogen Monoxide.

Ibrutinib primarily increases the risk of AF through its off-target effect on the C-terminal Src kinase (CSK) (Xiao et al., 2020). In a cardiomyocyte specific *CSK* knockout mouse model, Xiao et al. demonstrated increased rates of AF, left atrial enlargement, myocardial fibrosis, and inflammation with preserved ejection fraction (Xiao et al., 2020). Additionally, the *CSK* knockout mice exhibited a cytokine profile akin to that observed in mice treated with ibrutinib, along with a comparable gene expression pattern, suggesting similarities in the mechanisms underlying *CSK* loss and the use of ibrutinib. The connection between the inhibition of CSK and AF remains unclear, but it may be associated with downstream effects on the NOD-like receptor thermal protein domain associated protein 3 (NLRP3) inflammasome (Figure 2) (Awan et al., 2020). Long-term use of Ibrutinib can inhibit CSK from phosphorylating the carboxyl terminal of Src family kinases (SFK), which leads to the enhancement of SFK activity, atrial fibrosis, and activation of inflammatory reactions, thus increasing the susceptibility to AF (Figure 2). In addition to the CSK-mediated pathway, ibrutinib has also been demonstrated to elevate the expression of calmodulin kinase 2 (CaMK II) and enhance the phosphorylation of ryanodine receptor 2 (RyR 2) within the cardiomyocyte endoplasmic reticulum (Figure 2). This process impairs intracellular calcium management and initiates ectopic electrical activity (Awan et al., 2020).

The mechanism by which ibrutinib induces hypertension is not well understood (Figure 2). One potential mechanism involves its effects on vascular endothelial growth factor (VEGF). Ibrutinib has been demonstrated to reduce the secretion of homeostatic chemokines Chemokine C-X-C motif ligand 12 (CXCL12), CXCL13, and C-C Motif Chemokine Ligand 19 (CCL19), as well as VEGF in human cell lines, leading to decreased nitric oxide production and increased vascular tone (Pandey et al., 2018; Ping et al., 2017). Vascular remodeling and the production of inflammatory cytokines, resulting from the downregulation of the PI3K pathway mediated by BTK and TEC, have also been proposed as potential mechanisms (Liu N. et al., 2013; McMullen et al., 2014).

Ibrutinib-induced ventricular arrhythmias (VAs) are likely mediated by acute dysregulation of cardiomyocyte calcium handling and repolarization dynamics (Figure 2) (Kudinov and Darbar, 2020). In a hypertensive rat model, Du and colleagues demonstrated that acute treatment with ibrutinib induced ventricular fibrillation in older rats. This was achieved through enhanced action potential duration alternans and spatial discordance, a lower calcium alternans ratio, and a shorter time to peak calcium amplitude. In contrast, ibrutinib did not induce these changes or trigger ventricular arrhythmias in younger rats (Du et al., 2020). The molecular pathways through which ibrutinib induces these alterations remain inadequately understood.

#### 3.1.2 Acalabrutinib (ACP-196)

Acalabrutinib (ACP-196) (Table 1) is a second-generation, selective, irreversible inhibitor of BTK that has improved pharmacologic features, including favorable plasma exposure, rapid oral absorption, a short half-life, and the absence of irreversible targeting to alternative kinases, such as epidermal growth factor receptor (EGFR), TEC, and ITK (Byrd et al., 2016). In kinase-inhibition assays, acalabrutinib was a more selective BTK inhibitor than ibrutinib (Byrd et al., 2016). Unlike ibrutinib, acalabrutinib did not inhibit EGFR, ITK, or TEC (Byrd et al., 2016). In the *in vitro* assays, it was clearly demonstrated that, unlike ibrutinib, acalabrutinib had no effect on the phosphorylation of EGFR at tyrosine residues Y1068 and Y1173. At a concentration of 1,000 nM, ibrutinib completely suppressed TEC activity, whereas acalabrutinib at the same concentration exhibited minimal activity on TEC (Byrd et al., 2016). Compared to ibrutinib, acalabrutinib exhibits a significantly higher IC50 (>1,000 nM) and essentially no inhibition of kinase activities for ITK, EGFR, ERBB2, ERBB4, JAK3, BLK, FGR, FYN, HCK, LCK, LYN, SRC and YES1 (Byrd et al., 2016; Christensen et al., 2022).

In two separate pooled safety analyses of acalabrutinib monotherapy trials for a variety of B cell malignancies totaling 1802 patients, the incidence of AF was 4% (Brown et al., 2020; Furman et al., 2021). The majority of patients who developed AF in these trials had preceding cardiovascular risk factors prior to starting acalabrutinib. In both analyses, the median time to AF occurrence was 521 days and in contrast to ibrutinib, the incidence did not increase over time (Brown et al., 2020; Furman et al., 2021). No patients in the acalabrutinib arm discontinued therapy due to AF compared to 3.4% of patients in the ibrutinib arm (Byrd et al., 2021). Hypertension has been reported in 5%–9% of patients in clinical trials of acalabrutinib monotherapy (Brown et al., 2020; Byrd et al., 2021; Furman et al., 2021; Owen et al., 2020). No patients in the acalabrutinib group discontinued therapy due to hypertension. Although the rates of hypertension are relatively lower with acalabrutinib compared to ibrutinib, the increased incidence of hypertension in acalabrutinib trials suggests that hypertension could be an effect associated with the BTKi class (Table 2).

The mechanism of acalabrutinib-induced AF is not well understood. Unlike ibrutinib, acalabrutinib does not inhibit CSK in mouse models (Xiao et al., 2020). Furthermore, acalabrutinib does not effectively inhibit other kinases associated with hypertension, such as EGFR or TEC (Estupiñán et al., 2021). Acalabrutinib-induced AF is primarily attributed to its impact on cardiomyocyte intracellular calcium management, specifically through the modulation of CaMK Ⅱ and RyR pathways.

Like the hypertension caused by ibrutinib, the mechanism of hypertension linked to acalabrutinib remains not well understood. As it exerts a similar effect on TEC kinase as ibrutinib, it is plausible that the downstream PI3K downregulation as a result of TEC inhibition may alter the vascular endothelium via decreased nitrous oxide production (Ghia et al., 2020; McMullen et al., 2014). Further studies are needed to elucidate the underlying mechanism, which may uncover novel pathways involved in the development of hypertension.

#### 3.1.3 Zanubrutinib (BGB-3111)

Zanubrutinib (BGB-3111) is a second-generation irreversible BTKi (Table 1). Compared to ibrutinib, zanubrutinib exhibits heightened selectivity for BTK over ITK, leading to reduced inhibition of antigen-dependent cell-mediated cytotoxicity *in vitro*. However, it shows lower overall kinase selectivity for BTK when compared to acalabrutinib or tirabrutinib (Bond and Woyach, 2019). Zanubrutinib also inhibits off-target kinases, including EGFR, ITK, BMX, and the relaxin-Tec tyrosine kinase protein (RLX-TXK) (Estupiñán et al., 2021; Lipsky and Lamanna, 2020).

In the Phase Ⅲ ASPEN trial, which compared zanubrutinib to ibrutinib in 201 patients with Waldenstrom’s macroglobulinemia, AF was reported in only 2% of patients treated with zanubrutinib. There were no grade 3 AF events in the zanubrutinib arm and it was not discontinued for AF in any patient. The ASPEN study revealed hypertension incidence at 11% (including 6% at grade ≥3) among patients receiving zanubrutinib (Tam et al., 2020). The incidence and severity of most BTK-related toxicities, including AF, were lower with zanubrutinib than with ibrutinib (Tam et al., 2020). The treatment with zanubrutinib was associated with a trend towards improved response quality and reduced toxicity, particularly cardiovascular toxicity (Tam et al., 2020). No cases of VAs or sudden cardiac deaths have been reported in published clinical trials or post-marketing studies of zanubrutinib to date (Table 2) (An et al., 2021; Song et al., 2020; Tam et al., 2020).

#### 3.1.4 Tirabrutinib (Velexbru®)

Tirabrutinib (Velexbru®) is another covalent inhibitor currently approved only in Japan (Table 1). It is an oral medication that specifically targets and inhibits BTK by forming a covalent bond with C481, effectively blocking BCR signaling and thereby reducing the proliferation of malignant B cells. The Pharmaceuticals and Medical Devices Agency (PMDA) granted approval in 2020 for the treatment of relapsed/refractory primary central nervous system lymphoma (R/R PCNSL) (Dhillon, 2020; Gupta et al., 2025). Tirabrutinib exhibits a kinome profile analogous to acalabrutinib, characterized by high specificity for BTK, moderate inhibition of TEC, and negligible activity against EGFR, ERBB2-HER2, ITK, and JAK3 (Estupiñán et al., 2021). By the end of 2023, a total of 18 patients were enrolled in 9 hospitals in Taiwan, among whom atrial fibrillation was found (Liao et al., 2024). In addition, no major cardiovascular adverse events were reported.

#### 3.1.5 Orelabrutinib

Orelabrutinib is another highly potent, orally administered, covalent BTK inhibitor that exhibits strong selectivity for BTK, minimizing off-target effects (Table 1). In 2020, it received approval from China’s National Medical Products Administration (NMPA) for the treatment of patients with relapsed/refractory chronic lymphocytic leukemia (R/R CLL) or small lymphocytic lymphoma (SLL), as well as relapsed/refractory mantle cell lymphoma (R/R MCL) who had previously undergone at least one prior treatment (Deng et al., 2023; Gupta et al., 2025). In 2023, orelabrutinib expanded its therapeutic application and was approved in China for the treatment of relapsed/refractory marginal zone lymphoma (R/R MZL). Furthermore, the U.S. Food and Drug Administration (FDA) granted the drug a breakthrough therapy designation (BTD) for the treatment of R/R MCL. In published pivotal clinical trials and real-world studies, the incidence of atrial fibrillation is extremely low (usually reported as 0% or <1%) (Cao et al., 2023; Xu W. et al., 2019).

#### 3.1.6 Pirtobrutinib

Pirtobrutinib (Jaypirca®) is a highly selective, orally administered, non-covalent BTK inhibitor that received accelerated FDA approval in January 2023 for the treatment of R/R MCL after at least two prior lines of therapy, including a previous BTKi (Table 1) (Gupta et al., 2025; Keam, 2023; Wang et al., 2023). Grade ≥3 treatment-emergent adverse events (TEAEs) of hemorrhage (3.7%) and atrial fibrillation/flutter (1.2%) were relatively infrequent (Wang et al., 2023). Of the 323 patients in the trial, only 2 developed AF, both of whom had a history of AF prior to the study’s initiation. 5% (1% grade 3) of patients developed hypertension during the study, with a median follow-up of 6 months. Notably, among the 15 patients who discontinued a previous BTKi due to cardiotoxicity, none experienced a recurrent cardiac adverse event while taking pirtobrutinib (Table 2) (Mato et al., 2021). The incidence of cardiovascular toxicity (particularly atrial fibrillation and hypertension) of Pirtobrutinib is significantly lower than that of traditional BTK inhibitors such as Ibrutinib (Shah et al., 2024).

#### 3.1.7 Fenebrutinib

Fenebrutinib is an orally administered BTKi designed for the treatment of autoimmune diseases and lymphoma, with reduced off-target activity (Table 3). Unlike covalent BTK inhibitors such as ibrutinib and acalabrutinib, fenebrutinib binds to BTK non-covalently, enabling it to target both wild-type BTK and BTK with C481S mutations, a common resistance mechanism to first-generation covalent inhibitors (Crawford et al., 2018; Gupta et al., 2025). No cases of AF or hypertension were reported among the 307 study patients who received fenebrutinib, although only adverse events occurring at a frequency of 5% or greater were documented (Cohen et al., 2020), indicating that it has no obvious cardiovascular toxicity signal.

**TABLE 3:** Research status of BTK inhibitors in the clinical trials.

Drug	Characteristic(s)	Disease(s)	R&D progress and R&D company	IC50 (nM)					References(s)
				BTK	ITK	TEC	BMX	TXK	
Branebrutinib (BMS-986195)	Covalent (Irreversible) BTKi	SLE, RA	Phase Ⅰb Bristol-Myers Squibb	0.1	100	0.9	1.5	5	Watterson et al. (2019)
Nemtabrutinib (ARQ-531) (MK-1026)	Non-covalent (Reversible) BTKi Target both wild-type and C481S mutant BTK	R/R CLL, NHL, WM (Phase I/II), CLL, SLL (Phase III)	Phase Ⅲ Merck	0.85	>10,000	5.8	5.2	36	Woyach et al. (2022), Woyach et al. (2024)
Spebrutinib (CC-292/AVL292)	Covalent (Irreversible) BTKi Highly selective, covalent oral small-molecule	CLL/SLL, B cell NHL, WM(Phase I)	Phase Ⅱ Celgene/Avila Therapeutics	2.3	24	16	1.6	9.1	Brown et al. (2016), Evans et al. (2013)
Evobrutinib (M2951, MSC-2364447C)	Covalent (Irreversible) BTKi	MS (Phase II), SLE, MS (Phase III)	Phase Ⅲ Merck	8.9	NA	7,300	20	NA	Haselmayer et al. (2019)
Vecabrutinib (SNS-062)	non-covalent (Reversible) BTKi	R/R CLL, other B cell malignancies (Phase Ib/II)	Phase Ⅰb/Ⅱ Not to proceed with phase Ⅱ studies	3	14	14	224	474	Jebaraj et al. (2022)
Fenebrutinib (GDC-0853)	non-covalent (Reversible) BTKi Less off-target activity	R/R B-NHL and CLL (Phase I), RA, SLE, CSU, MS (Phase II)	Phase Ⅲ Genentech	2.3	>1,000	>1,000	351	>1,000	Crawford et al. (2018)
Remibrutinib (LOU064)	Covalent (Irreversible) BTKi	CSU, MS	Phase Ⅱ Novartis	1.3	NA	NA	NA	NA	Lin et al. (2024)
Poseltinib (HM-71224)	Covalent (Irreversible) BTKi	Refractory CNSL, DLBCL, PCNSL, RA	Phase Ⅱ Hanmi Pharmaceutical/Eli Lilly	1.95	103	4.57	0.64	4.62	Park et al. (2016), Byun et al. (2021)
Elsubrutinib (ABBV-105)	An orally active, potent, selective and irreversible BTKi	Inflammatory disease	Phase Ⅱ AbbVie, Inc.	0.18	NA	NA	NA	NA	Goess et al. (2019)
Edralbrutinib (EBI-1459 SHR-1459 TG-1701)	An orally available, irreversible BTKi	B-NHL, CLL	Phase Ⅱ Eternity Bioscience, Inc.	6.7	NA	NA	NA	NA	Ribeiro et al. (2021)
Rilzabrutinib (PRN1008)	Covalent (Irreversible) BTKi	ITP, immune-mediated diseases	Phase Ⅲ Sanofi/Principia Biopharma	1.3	440	0.8	1.0	1.2	Langrish et al. (2021)
Tolebrutinib (SAR442168, PRN2246)	Covalent (Irreversible) BTKi	MS	Phase Ⅲ Sanofi/Principia Biopharma	0.7	NA	NA	NA	NA	Owens et al. (2022)
BMS-935177	A potent and selective reversible BTKi	Autoimmune diseases	Phase Ⅰ Bristol Myers Squibb Co.	3.0	96	13	24	180	De Lucca et al. (2016)

SLE, systemic lupus erythematosus; RA, rheumatoid arthritis; R/R CLL, relapsed/refractory chronic lymphocytic leukemia; NHL, non-hodgkin lymphoma; WM, Waldenström’s macroglobulinemia; CLL, chronic lymphocytic leukemia; SLL, small lymphocytic lymphoma; MS, multiple sclerosis; RA, rheumatoid arthritis; CSU, chronic spontaneous urticaria; CNSL, central nervous system lymphoma; DLBCL, diffuse large B cell yymphoma; PCNSL, primary central nervous system lymphoma; B-NHL, B cell non-Hodgkin lymphoma; ITP, immunologic thrombocytopenic purpura; NA, no data.

#### 3.1.8 Other BTKi

Other BTK inhibitors are summarized in Tables 3, 4. Branebrutinib (BMS-986195) is a potent, highly selective, oral small-molecule covalent BTKi (Catlett et al., 2020; Watterson et al., 2019). Evobrutinib is also a covalent BTKi that effectively inhibits BTK activity in both B cells and myeloid cells, both of which are pivotal in mediating immune responses. By targeting these pathways, evobrutinib aims to reduce inflammation and autoimmunity (Caldwell et al., 2019; Haselmayer et al., 2019). Spebrutinib is a highly selective, covalent oral small-molecule BTKi that targets the same C481 residue in BTK as ibrutinib, thereby blocking BTK signaling. Preclinical studies demonstrated that spebrutinib effectively inhibited B cell activation dependent on BCR (Brown et al., 2016). Nemtabrutinib, previously known as ARQ-531, is a noncovalent BTKi designed to target both wild-type (WT) and C481S mutant BTK, including resistance mutations. It is being explored as a treatment for R/R CLL/SLL and other hematological malignancies (Woyach et al., 2022). Vecabrutinib is another orally administered noncovalent BTKi designed to target both WT BTK and BTK with the C481S mutation, a common mutation that confers resistance to ibrutinib and acalabrutinib. Vecabrutinib functions by inhibiting the phosphorylation of BTK, thereby blocking the activity of its downstream target, phosphatidylinositol-specific phospholipase Cγ2 (PLCγ2). Despite generally being well tolerated, the lack of efficacy and observed adverse effects led to the decision not to proceed with Phase II studies (Cohen et al., 2020).

**TABLE 4:** Research status of BTK inhibitors in the preclinical trials.

Drug	Characteristic(s)	Disease(s)	R&D progress and R&D company	IC50 (nM)					References(s)
				BTK	ITK	TEC	BMX	TXK	
RN-486	Non-covalent (Reversible) BTKi	RA, SLE	Pre-clinical F. Hoffmann-La Roche	4.0	NA	NA	NA	NA	Xu et al. (2012)
GDC-0834	Non-covalent (Reversible) BTKi	RA	Pre-clinical Genentech/Gilead	5.9	NA	NA	NA	NA	Liu et al. (2011)
CGI-1746	Non-covalent (Reversible) BTKIs	RA	Pre-clinical CGI Pharmaceuticals	1.9	4,270	>10,000	1870	NA	Di Paolo et al. (2011)
CNX-774	Covalent (Irreversible) BTKi	NA	Pre-clinical Avila Therapeutics	<1.0	NA	NA	NA	NA	Akinleye et al. (2013)
JS25	A selective and covalent BTKi	CLL	Pre-clinical	5.8	440	220	49	190	Sousa et al. (2022)

RA, rheumatoid arthritis; SLE, systemic lupus erythematosus; CLL, chronic lymphocytic leukemia; NA, no data.

### 3.2 BMX inhibitors

In contrast to BTK, the development of inhibitors for other TEC kinases has been relatively limited, despite considerable evidence indicating their significant roles in hematopoiesis and their potential as therapeutic targets (Forster et al., 2020). Table 5 summarizes the related contents of BMX inhibitors in previous studies.

**TABLE 5:** Research status of BMX, ITK, TXK and TEC inhibitors.

Drug	Characteristic(s)	Disease(s)	R&D progress and R&D company	IC50 (nM)					References(s)
				BTK	ITK	TEC	BMX	TXK	
BMX-IN-1	A potent, selective, and irreversible BMX kinase inhibitor	Prostate cancer	Pre-clinical	10.4	5,250	653	8	377	Liu F. et al. (2013)
CHMFL-BMX-078	A highly selective type II irreversible BMX inhibitor	Melanoma	Pre-clinical	437	NA	NA	11	NA	Liang et al. (2017), Jiang et al. (2022)
IHMT-15130	A highly potent and selective irreversible BMX inhibitor	Cardiac hypertrophy	Pre-clinical	NA	NA	NA	1.47	NA	Qi et al. (2025)
Soquelitinib (CPI-818)	An orally active and highly selective covalent ITK inhibitor	T Cell Lymphoma	Phase 1b/2 Akros Pharma, Inc.	NA	NA	NA	NA	NA	Hsu et al. (2024)
JTE-051	A selective ITK inhibitor	Immune system diseases	Phase II Angel Pharmaceuticals Co., Ltd.	NA	NA	NA	NA	NA	Zarrin et al., (2021), Study to Evaluate Efficacy (2025)
PRN694	Irreversible, highly selective and potent covalent ITK and RLK dual inhibitor	Psoriasis, autoimmune, inflammatory, malignant diseases	Pre-clinical	17	0.3	3.3	17	1.4	Zhong et al. (2015), Cho et al. (2015)
BMS-509744	Potently and selectively inhibits ITK kinase activity	Psoriasis	Pre-clinical	4,100	19	17,000	>50,000	11,000	Lin et al. (2004)
BSJ-05–037	A potent and selective heterobifunctIonal degrader of ITK	Psoriasis	Pre-clinical	NA	17.6	NA	NA	NA	Jiang et al. (2023)
Acalabrutinib/Calquence (ACP-196)	The selectivity of TXK is 19 times lower than that of BTK, but there is still cross-inhibition.	Untreated or refractory CLL/SLL, pretreated MCL	Approved by FDA and EMA in 2017 AstraZeneca/Acerta Pharma BV	5.1	>1,000	93	46	368	Byrd et al. (2016), Acalabrutinib, (2012), Markham and Dhillon (2018)
Ritlecitinib (PF-06651,600)	Irreversible inhibitor of JAK3 and TEC kinase family	AA, Vitiligo, UC	Approved by FDA and EMA in 2023 Pfizer Inc.	607	8,510	592	606	193	Thorarensen et al. (2017), Telliez et al. (2016)

CLL, chronic lymphocytic leukemia; SLL, small lymphocytic lymphoma; MCL, mantle cell lymphoma; AA, alopecia areata; UC, ulcerative colitis; NA, no data.

BMX-1N-1 is a potent, selective, and irreversible inhibitor of BMX kinase, which covalently modifies Cys496 (Liu F. et al., 2013). CHMFL-BMX-078 is a highly selective and potent type II irreversible inhibitor of BMX kinase. It demonstrated an IC50 of 11 nM by forming a covalent bond with the Cys496 residue in the DFG-out inactive conformation of BMX (Liang et al., 2017). IHMT-15130 is a potent and irreversible BMX kinase inhibitor that covalently targets cysteine 496 of BMX and exhibits potent inhibitory activity against BMX kinase. IHMT-15130 shows selectivity for CSK kinase, and its targeting of CSK may lead to severe atrial fibrillation and bleeding (Qi et al., 2025). All agents are in preclinical studies, and none has been reported to exhibit cardiovascular toxicity.

In the context of cardiovascular pathology, BMX has been reported to be upregulated in atherosclerotic plaques, models of myocardial ischemia-reperfusion injury, and heart failure models, potentially contributing to disease progression by exacerbating inflammation and driving pathological remodeling (Holopainen et al., 2015; Qi et al., 2025). Theoretically, therefore, BMX inhibition may impact the cardiovascular system by disrupting endothelial homeostasis, vascular repair processes, and myocardial adaptive responses. Currently, highly selective BMX inhibitors (e.g., BMX-IN-1, CHMFL-BMX-078) remain in the preclinical stage, with no systematic reports of cardiovascular toxicity to date (Jiang et al., 2022; Liang et al., 2017; Liu F. et al., 2013). Notably, the first-generation BTK inhibitor ibrutinib also acts as a potent BMX inhibitor (Table 1) (Burger and Buggy, 2013; Qiu et al., 2014). Whether the cardiovascular toxicities observed clinically with ibrutinib, particularly hypertension, which may involve endothelial dysfunction, are partially attributable to BMX inhibition represents an open question warranting further investigation (Shirley, 2022). This highlights that endothelial function, blood pressure, and cardiac remodeling should be considered key safety endpoints during the development of BMX-targeted or broad-spectrum TEC family kinase inhibitors.

### 3.3 ITK inhabitors

Soquelitinib (CPI-818) is an orally active and highly selective covalent ITK inhibitor and is a potential novel target to enhance the immunotherapy of cancer (Hsu et al., 2024). Currently, Phase 1b/2 clinical trials of this agent are underway in China. JTE-051 is another ITK inhibitor currently under clinical evaluation for the treatment of rheumatoid arthritis (RA) and psoriasis (Table 5) (Zarrin et al., 2021). PRN694 is a highly selective and potent covalent inhibitor of ITK and TXK (Table 5); its extended target residence time allows for durable attenuation of effector cells (Zhong et al., 2015). This ITK/RLK dual inhibitor was approved for the treatments of T-cell or NK cell malignancies as well as inflammatory and autoimmune diseases, such as psoriasis, psoriatic arthritis, rheumatoid arthritis, multiple sclerosis, and irritable bowel disease (Fuhriman et al., 2018; Zhong et al., 2015). BMS-509744 is a potent and selective ITK inhibitor which inhibits anti-TCR antibody induced IL-2 production (Table 5) (Das et al., 2006; Lo, 2010).

While the role of ITK in adaptive immunity is well-established (Lechner et al., 2020), direct evidence for its expression and function in cardiovascular cells remains limited. However, T cell-driven inflammation is a critical mechanism in atherosclerosis, myocarditis, and certain cardiomyopathies (Chen et al., 2023; Rosenzweig et al., 2021). Thus, inhibition of ITK may indirectly influence the progression of cardiovascular diseases, such as atherosclerosis, through systemic immunosuppression and modulation of the inflammatory cytokine network. Currently, ITK inhibitors in clinical development are primarily targeting T cell-mediated autoimmune diseases. According to publicly available data from these trials, no significant cardiovascular safety signals have been reported. Nonetheless, given the close link between inflammation and cardiovascular diseases, it remains a prudent measure to monitor cardiovascular events in patients receiving long-term ITK inhibitor therapy.

### 3.4 TXK inhabitors

Similar to ITK, TXK is directly involved in TCR signaling. To date, no highly selective TXK inhibitors have entered clinical development. Some multi-target inhibitors (e.g., PRN694) also exhibit activity against TXK (Zhong et al., 2015). Given its more restricted tissue expression, primarily limited to lymphoid lineages, and the scarcity of studies linking TXK to cardiovascular functions (Kashiwakura et al., 1999), it is generally considered that inhibiting TXK carries a low risk of direct cardiovascular toxicity (Yin et al., 2022). However, this conclusion still requires further verification through future studies employing specific pharmacological tools and more in-depth investigation.

### 3.5 TEC inhibitors

Ritlecitinib (LITFULO™) is a kinase inhibitor being developed by Pfizer for the treatment of alopecia areata, vitiligo, ulcerative colitis, and Crohn’s disease. Ritlecitinib irreversibly inhibits JAK3 and the TEC kinase (Table 5) (Blair, 2023; Xu H. et al., 2019). Among the 319 randomized patients, there was one death due to myocardial infarction, which was considered unrelated to the study drug (Sandborn et al., 2023). No major adverse cardiovascular events, opportunistic infections, or deaths were reported (Hordinsky et al., 2023; King et al., 2023; King et al., 2021). However, it is necessary to pay attention to its effects on the vascular endothelium (BMX inhibition) and the PI3K-AKT pathway, as these may increase the risk of bleeding or myocardial injury. In another study, within the all-exposure pool for the ritlecitinib 50-mg group, three patients (0.2%) with adjudicated Major Adverse Cardiovascular Events (MACE) as serious adverse events were reported (King et al., 2024; FDA, 2025). Notably, patients with cardiovascular risk factors were not specifically excluded from the ALLEGRO studies, and all patients who experienced cardiovascular events had at least one cardiovascular risk factor (King et al., 2024). Thrombosis has occurred in patients treated with LITFULO (FDA, 2025). The relationship between thromboembolic and cardiovascular events and the increased lipid levels observed with some JAK inhibitors is not well understood (Gladman et al., 2019).

In the cardiovascular context, TEC plays a role in VEGF and shear stress-induced endothelial cell signaling, potentially affecting angiogenesis and endothelial barrier function (Klüppel et al., 1997). Furthermore, TEC is also involved in platelet activation signaling (Atkinson et al., 2003). Therefore, inhibition of TEC may potentially influence vascular integrity, thrombus formation, and vascular remodeling processes. Although preliminary clinical cardiovascular safety data for ritlecitinib indicate a relatively low risk, theoretical concerns remain regarding its impact on vascular endothelium and the PI3K-AKT pathway, which may be associated with bleeding tendency or alterations in vascular function (Herrmann, 2020). Inhibition of TEC by BTK inhibitors such as ibrutinib (Table 1) may also contribute to their toxicity profile. In the future, developing more selective TEC inhibitors combined with refined cardiovascular function assessments will help elucidate the independent contribution of TEC inhibition to cardiovascular toxicity.

## 4 Conclusion

TFKis represent a class of transformative therapeutics, yet their associated cardiovascular toxicity remains a significant clinical challenge (Table 6). This review synthesizes current evidence, yielding several key insights. First, cardiovascular toxicity is a well-established class effect of BTKis, with its incidence and severity inversely correlating with kinase selectivity; AF and hypertension are the most frequently observed events (Quartermaine et al., 2023). Second, the underlying mechanisms arise from a combination of on-target BTK inhibition and off-target inhibition of other kinases such as CSK and TEC. Key implicated pathways include dysregulated calcium handling (CaMKII/RyR2), inflammation (NLRP3), fibrosis, and endothelial dysfunction (VEGF/PI3K-NO) (Kudinov and Darbar, 2020; N. Liu et al., 2013; McMullen et al., 2014; Pandey et al., 2018; Ping et al., 2017). While the cardiovascular toxicity of BTK inhibitors has garnered substantial attention, future research urgently needs to extend to inhibitors targeting other TEC family kinases and long-term cardiovascular safety data for inhibitors specifically targeting other TFK members remain scarce. Regarding BMX, highly selective inhibitors should be developed and systematically evaluated in models such as atherosclerosis and myocardial hypertrophy to assess their effects on vascular endothelium and myocardial remodeling. For ITK, it is necessary to clarify its role in T cell-mediated cardiovascular inflammation (e.g., atherosclerosis) and to explore the balance between its immunomodulatory effects and cardiovascular safety. Although TXK has limited direct cardiovascular actions, its immunomodulatory potential still requires validation through specific tool compounds. Concerning TEC, long-term clinical follow-up should focus on endothelial function, platelet activation, and the PI3K-AKT pathway, particularly in populations at high cardiovascular risk.

**TABLE 6:** Summary of cardiotoxicity of TEC family kinase inhibitors.

Drug	Toxicity Phenotype	Proposed Mechanism(s)	Key Evidence
Ibrutinib (1st-gen covalent BTKi)	AF (5%–16% incidence)	Off-target inhibition of C-terminal Src kinase (CSK) → increased Src family kinase (SFK) activity → atrial fibrosis and inflammation via NLRP3 inflammasome. Altered cardiomyocyte Ca2+ handling via CaMKII/RyR2 pathway.	Clinical trials and real-world analyses show AF leading to drug discontinuation in 20%–60% of cases where it occurs (Maddocks et al., 2015; Wiczer et al., 2017). Mouse model with cardiomyocyte-specific CSK knockout recapitulates AF phenotype (Xiao et al., 2020).
	Hypertension (up to 80% in real-world studies)	Reduced secretion of VEGF and homeostatic chemokines → decreased nitric oxide (NO) production → increased vascular tone. Downregulation of PI3K pathway via BTK/TEC inhibition may contribute to vascular remodeling.	Reported in up to 30% of patients in trials, up to 80% in real-world studies (Dickerson et al., 2019). Retrospective analysis shows increased risk vs. chemotherapy (Abdel-Qadir et al., 2021).
Ibrutinib (1st-gen covalent BTKi)	VAs and HF	Acute dysregulation of cardiomyocyte Ca^2+^ handling and repolarization. Long-term mechanisms may involve pro-fibrotic and inflammatory pathways.	Case reports and retrospective analyses link ibrutinib to VAs (Guha et al., 2018; Kudinov and Darbar, 2020). Long-term follow-up data indicate up to 5% HF incidence (Barr et al., 2022; Munir et al., 2019); 3-year risk of 7.7% in one analysis (Abdel-Qadir et al., 2021). Hypertensive rat model shows acute induction of ventricular fibrillation (Du et al., 2020).
Acalabrutinib (2nd-gen covalent BTKi)	AF (∼4% incidence)	Primarily attributed to impact on cardiomyocyte intracellular Ca2+ management (CaMKII/RyR pathways). Does not significantly inhibit CSK.	Pooled safety analyses of trials (n = 1802) report 4% AF incidence, median time to event 521 days, no increase over time (Brown et al., 2020; Furman et al., 2021). Lower discontinuation rate due to AF vs. ibrutinib (Byrd et al., 2021).
	Hypertension (5%–9% incidence)	Mechanism not fully understood. Similar to ibrutinib, potential role of TEC inhibition and downstream PI3K downregulation affecting vascular endothelium and NO production.	Reported in clinical trials (Brown et al., 2020; Byrd et al., 2021; Furman et al., 2021; Owen et al., 2020). Incidence suggests it may be a class effect, but less frequent/severe than with ibrutinib.
Zanubrutinib (2nd-gen covalent BTKi)	AF (∼2% incidence)	Not explicitly detailed, but improved selectivity profile (over ITK, *etc.*) likely reduces off-target toxicity.	In ASPEN trial vs. ibrutinib, only 2% AF reported (0% grade 3, no discontinuations) (Tam et al., 2020).
	Hypertension (11% incidence, 6% ≥grade 3)	Mechanism not specified, but off-target kinase profile differs from ibrutinib.	Reported in ASPEN trial (Tam et al., 2020).
Pirtobrutinib (Non-covalent BTKi)	Atrial Fibrillation/Flutter (1.2% grade ≥3)	Mechanism not studied. Hypothesized that reversible, non-covalent binding and high selectivity may minimize off-target effects.	In BRUIN trial (n = 323), only 2 patients developed AF (both had prior history) (Mato et al., 2021; Wang et al., 2023). No recurrent cardiac events in patients who discontinued prior BTKi due to cardiotoxicity (Mato et al., 2021).
	Hypertension (5% incidence, 1% grade 3)	Mechanism not specified.	Reported in BRUIN trial with 6-month median follow-up (Mato et al., 2021; Wang et al., 2023).
Fenebrutinib (Non-covalent BTKi)	No significant signal for AF or hypertension reported	High selectivity for BTK, minimal off-target activity.	In a phase II RA trial (n = 307), no cases of AF or hypertension were reported among documented AEs (≥5% frequency) (Cohen et al., 2020).
Ritlecitinib (JAK3/TEC inhibitor)	MACE (low incidence)	Mechanism not fully elucidated. Potential concern due to TEC inhibition affecting vascular endothelium (PI3K-AKT pathway) and platelet function.	In clinical trials for alopecia areata, low rates of adjudicated MACE reported (0.2% in 50-mg group). All patients with events had pre-existing CV risk factors (King et al., 2024; FDA, 2025). One unrelated death due to MI reported (Sandborn et al., 2023).
BMX Inhibitors (e.g., BMX-IN-1, CHMFL-BMX-078)	No systematic cardiovascular toxicity reported (preclinical stage)	Theoretically, BMX inhibition could disrupt endothelial homeostasis and vascular repair, given its role in atherosclerosis and myocardial remodeling models.	All are preclinical; no clinical CV toxicity data (Liang et al., 2017; Liu F et al., 2013). Ibrutinib’s potent BMX inhibition raises the question of its contribution to ibrutinib’s hypertension/endothelial toxicity (Shirley, 2022).
ITK Inhibitors (e.g., Soquelitinib, JTE-051, PRN694)	No significant cardiovascular safety signals reported in current trials.	Primary action is immunomodulation via T-cells. May indirectly influence CVD progression (e.g., atherosclerosis) via systemic suppression of T cell-driven inflammation.	Clinical trials for autoimmune diseases; no major CV events highlighted in available data (Hsu et al., 2024; Zarrin et al., 2021; Zhong et al., 2015). Theoretical risk due to inflammation-CVD link warrants monitoring.
TXK Inhibitors	Low presumed risk of direct cardiovascular toxicity	Limited tissue expression (primarily lymphoid). No strong evidence linking TXK to cardiovascular function.	No highly selective TXK inhibitors in clinical development. Some multi-target inhibitors (e.g., PRN694) have TXK activity (Zhong et al., 2015).

AF, atrial fibrillation; BTKi, Bruton’s Tyrosine Kinase Inhibitor; CaMKII, Calcium/calmodulin-dependent protein kinase II; CSK, C-terminal Src kinase; CV, cardiovascular; CVD, cardiovascular disease; MACE, major adverse cardiovascular events; MI, myocardial infarction; NLRP3, NOD-like receptor thermal protein domain associated protein 3; NO, nitric oxide; PI3K, Phosphatidylinositol 3-kinase; RyR2, Ryanodine Receptor 2; SFK, Src family kinase; TEC, tyrosine kinase expressed in hepatocellular carcinoma; VEGF, vascular endothelial growth factor.

Given their distinct tissue distributions and functions, these agents may present different risk profiles, a possibility that urgently warrants targeted investigation. From a clinical management standpoint, important strategies include opting for newer, more selective agents, conducting proactive cardiovascular risk assessments, implementing enhanced monitoring, and fostering multidisciplinary care within a cardio-oncology framework.
